# Plasma Activation of Polyethylene Powder

**DOI:** 10.3390/polym12092099

**Published:** 2020-09-15

**Authors:** Hana Šourková, Petr Špatenka

**Affiliations:** Faculty of Mechanical Engineering, Department of Materials Engineering, Czech Technical University in Prague, Karlovo náměstí 13, 121 35 Prague 2, Czech Republic; hana.sourkova@fs.cvut.cz

**Keywords:** polymer powder, surface activation, plasma treatment, batch mode treatment

## Abstract

Polyethylene powder of average particle diameter of 160 µm was activated in a plasma reactor made from aluminum of volume 64 dm^3^ at the pressure 100 Pa. Dense oxygen plasma was sustained with a microwave discharge powered by a pulsed magnetron source of power 1 kW mounted onto the top flange of the plasma reactor. Polymer powder was treated in a batch mode with 0.25 kg/batch. The powder was placed into a stainless-steel dish mounted in the center of the reactor where diffusing plasma of low ion density, and the O-atom density of 2 × 10^21^ m^−3^ was sustained. The powder was stirred in the dish at the rate of 40 rpm. The evolution of powder wettability versus treatment time was measured using the Washburne method, and the surface composition was determined by X-ray Photoelectron Spectroscopy (XPS). The wettability versus the oxygen concentration assumed a parabolic behavior. The maximal oxygen concentration, as revealed by XPS, was 17.5 at.%, and the maximal increase of wettability was 220%. The efficiency of O-atoms utilization in these experimental conditions was about 10% taking into account the spherical geometry of dust particles and perfectly smooth surface. The method is scalable to large industrial systems.

## 1. Introduction

Polyethylene (PE) is a low-melting-point polyolefin that consists of nonpolar, saturated, high molecular weight hydrocarbons. Due to easiness of handling and the low price, it is the most commonly used plastic. It exhibits a low electrical conductivity, so it is suitable for insulation elements between the electrodes. It is chemically inert and thus useful in numerous applications. The mechanical properties are often inadequate, so it is reinforced by various fillers, often organic or inorganic fibers. Similar to other polyolefins, polyethylene is moderately hydrophobic material with a water contact angle (WCA) of about 90°. Its composition and structure prevent interaction with most other materials, therefore, the surface properties should be modified to assure for reasonable adhesion of any material connected to a product made from PE [1]. Nowadays, a standard technique for increasing the surface energy and thus increasing the adhesion properties is a brief treatment with gaseous plasma. A piece of PE is exposed to plasma rich in oxidizing radicals such as O, OH, N, NO, etc. and the radicals interact chemically on the PE surface, forming a variety of polar functional groups. PE foils have been treated by various authors using different setups, but they all report on increased wettability upon exposure to plasma sustained in different gases, including noble gases (usually argon), oxygen, air, water vapor and more complex gases [2,3,4,5,6,7]. The treatment times adopted by different authors varied from few s to several minutes, but the final wettability was always moderate at the water contact angle of around 35°. Super-hydrophilic surface finish (WCA below a few degrees) of PE has never been reported in scientific literature, indicating that the combined effect of saturation with highly polar functional groups and rich morphology on a sub-micrometer scale has never been obtained for this particular polymer. However, the achievable hydrophilicity was found good enough in many practical applications [8].

More challenging than surface activation of foils or similar materials is the activation of polymer powder. The powder is chemically equivalent to the foils, but the surface-to-mass ratio is much larger. The size of the powder particles prevents their assembling in the form of a monolayer that would assure for uniform treatment of all particles. If a much thicker powder layer were assembled onto a sample holder and treated by gaseous plasma, the powder on the surface would be over-treated, and the powder deep below the surface hardly influenced by plasma treatment. The non-sufficient exposure to gaseous plasma will lead to poor surface functionalization and thus inadequate wettability, and the over-treatment usually leads to softening, agglomeration and similar thermal effects. Various solutions have appeared to overcome the problem of inhomogeneous treatment. A comprehensive review was published recently by Arpagaus et al. [9]. The possible solutions include the fluidization [10,11], rotation [12], vibration and conveying [13] and stirring [14,15]. All these techniques allow for mixing the powder upon plasma treatment and enable reasonably uniform treatment of all particles in a batch.

## 2. Theoretical Limitations

Theoretically, the ideal treatment conditions will be met by levitating separate polymer powder particle in gaseous plasma. Suppose gaseous plasma is uniform in the vicinity of a powder particle levitating in the non-equilibrium gas. The reactive particles will collide with the polymer particle and cause different surface reactions. Many are exothermic so that they will lead to particle heating. The heating rate depends on the flux of the reactive particles onto the surface of the polymer particle. The flux of neutral reactive particles is

(1) $$j_{N}=1/4\times n_{N}\langle v_{N}\rangle$$

where $n_{N}$ is the density of neutral reactive particles in the vicinity of the polymer particle and $\langle v_{N}\rangle$ is the average random velocity of the neutral particles. If the neutral reactive particles are oxygen atoms, $\langle v_{N}\rangle$ is 630 m/s at room temperature. The heating power as the consequence of heterogeneous surface recombination of atoms to parent molecules depends on the recombination coefficient:

(2) $$P_{N}=j_{N}\cdot S_{\mathrm{powder}}\cdot \gamma \cdot \left(W_{D}/2\right)$$

where *S*_powder_ is the surface of the powder particle. The polymer particles are often fairly spherical, so the surface is calculated as for an ideal sphere, i.e., *S*_powder_ = 4π r^2^. The factor *γ* in Equation (2) is the coefficient for heterogeneous surface recombination of atoms on the polymer surface, and *W*_D_/2 is half of the dissociation energy of the parent molecule. The dissociation energy for oxygen molecules is 5.2 eV, and the probability for heterogeneous surface recombination on a smooth polymer surface is around 10^−3^ [16].

The polymer particle levitating in a non-equilibrium gaseous plasma of the atom density $n_{N}$ is heated by heterogeneous surface recombination of atoms at the rate

(3) $$\frac{dT}{dt}=\frac{P_{N}}{\left(m\cdot c_{p}\right)}$$

where *m* is the mass of the particle (*m* = *ρV,* where *ρ* is the density of polymer material and *V* is the volume of the polymer particle) and *c*_p_ is the specific thermal capacity of the polymer material. Rearranging Equations (1)–(3) one can calculate the heating rate upon exposure of a polymer particle levitating in non-equilibrium gas rich in oxygen atoms as

(4) $$\frac{dT}{dt}=\frac{1/4\cdot n_{N}\cdot \langle v_{N}\rangle \cdot 4\pi r^{2}\cdot \gamma \cdot \left(W_{D}/2\right)}{\rho \cdot 4\pi r^{3}/3c_{p}}=\frac{3\cdot n_{N}\cdot \langle v_{N}\rangle \cdot \gamma \cdot W_{D}}{8\cdot \rho \cdot r\cdot c_{p}}$$

**Figure 1:**
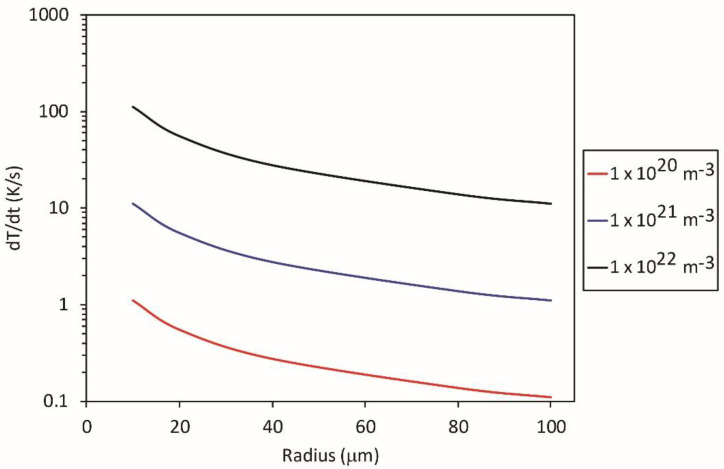
The heating of a spherical polymer particle levitating in non-equilibrium gas rich in O-atoms versus the particle radius. The density of O-atoms is the parameter.

Taking into account typical numerical values, i.e., neutral particles’ kinetic temperature of 300 K resulting in $\langle v_{N}\rangle$ = 630 m/s, *γ* = 10^−3^, *W*_D_ = 8.3 × 10^−19^ J, *ρ* = 940 kg/m^3^ and *c*_p_ = 2250 J/kg K, one can calculate the heating rate for various experimental conditions. Figure 1 represents the heating rate of polyethylene powder levitating in non-equilibrium gas versus the radius of the powder particles for three densities of O-atoms, i.e., 10^20^, 10^21^ and 10^22^ m^−3^. A typical O-atom density in a highly dissociated oxygen plasma sustained at a rather low discharge power is 10^21^ [17]. The heating is marginal for a moderate O-atom density but should be taken into account at high densities of oxygen atoms and low diameter of polymer particles.

Any macroscopic particle levitating in non-equilibrium gaseous plasma assumes the floating potential. This potential is negative against un-perturbed plasma and enables equilibration of the electron and positive ion fluxes onto the particle surface. In between, a sheath is formed. The electric field in the sheath retards all electrons except the fastest and accelerate all positively charged ions that have reached the sheath boundary onto the particle surface. The voltage across the sheath depends on the electron temperature and mass of positively charged ions. The sheath thickness increases with increasing electron temperature and decreases with the increasing electron density. The sheath thickness is of the order of the Debye length, which has been defined for highly non-equilibrium plasma useful for plasma treatment of polymer materials as

(5) $$\lambda_{D}=\sqrt{\frac{\varepsilon_{0}k_{B}T_{e}}{n_{e}e_{0}^{2}}}$$

where $\varepsilon_{o}$ is vacuum permittivity, $k_{B}$ is the Boltzmann constant, *T*_e_ is the electron temperature, $n_{e}$ is the electron density and $e_{0}^{}$ is the elementary charge. The sheath does not have a sharp boundary, so its thickness is not known. It is definitely larger than the Debye length. The Debye length and some other plasma parameters can be calculated using a suitable plasma calculator, for example the one provided by Bochum University [18]. The positively charged ions, which are almost perfectly thermal in unperturbed plasma at the temperature close to the kinetic temperature of neutral gas (often just above 300 K), accelerate in the pre-sheath and enter the sheath at Bohm velocity:

(6) $$v_{B}=\sqrt{\frac{k_{B}T_{e}}{M_{i}}}$$

where *T*_e_ is the electron temperature in gaseous plasma and *M*_i_ is the ion mass. All ions are accelerated towards a macroscopic particle and bombard the surface with the kinetic energy, which in the collision-less approximation corresponds to the voltage drop across the sheath. Furthermore, all ions which have entered the sheath also neutralize on the surface. The energy dissipated on the macroscopic particle surface is, therefore, the sum of ionization energy and kinetic energy gained within the sheath. For oxygen plasma at the electron temperature of 3–4 eV (very typical values), the energy is about *W*_i_ = 25 eV.

**Figure 2:**
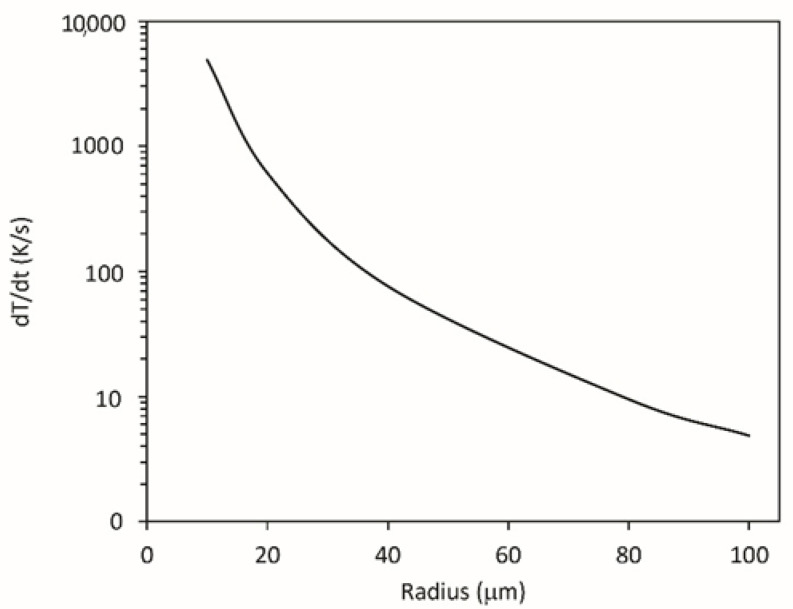
The heating of a spherical polymer particle levitating in non-equilibrium oxygen plasma versus the particle radius.

The flux of positively charged ions onto the surface of a macroscopic particle levitating in non-equilibrium gaseous plasma is

(7) $$j_{i}=n_{i}\langle v_{B}\rangle$$

where *n*_i_ is the density of positively charged ions in bulk plasma. If the ions are O_2_^+^, $\langle v_{B}\rangle$ is 4240 m/s at the electron temperature of 3 eV [18]. The heating power as the consequence of treatment by positively charged ions is:

(8) $$P_{i}=j_{i}\cdot S_{\mathrm{sheath}}\cdot W_{i}$$

where *S*_sheath_ is the surface of the sheath. There is a small but very important difference between Equations (2) and (8): there is the surface of a powder particle in Equation (2), while in Equation (8) it is the surface of sheath. For large objects (dimension orders of magnitude larger than the Debye length), the surfaces are almost identical, but for small powder they different significantly. For spherical geometry and assuming the sheath thickness of *λ*_D_ much larger than the polymer particle diameter, a macroscopic particle levitating in non-equilibrium gaseous plasma of the ion density *n*_i_ is heated at the rate

(9) $${\left(dT/dt\right)}_{\mathrm{ion}}=\frac{P_{i}}{m\cdot c_{p}}=\frac{3\cdot n_{i}\cdot \langle v_{B}\rangle \cdot r_{\mathrm{sheath}}^{2}\cdot W}{\rho \cdot r^{3}\cdot c_{p}}\approx \frac{3\cdot n_{i}\cdot \langle v_{B}\rangle \cdot \lambda_{D}^{2}\cdot W_{i}}{\rho \cdot r^{3}\cdot c_{p}}$$

where *r*_sheath_ and *r* are the radii of sheath and the polymer particle, respectively. The sheath thickness assumed in Equation (9) is equal to *λ*_D_. In practice, the sheath thickness is larger; therefore, Equation (9) represents the lower value of the heating rate. Figure 2 represents the heating rate of polyethylene powder levitating in non-equilibrium oxygen plasma with the neutral gas kinetic temperature of 300 K and the electron temperature of 3 eV versus the radius of the powder particles. The heating rate does not depend on the density of ions (*n*_i_), because the Debye length is inverse proportional to the square root of the ion density, as in Equation (5). The heating of powder particles levitating in plasma by ions is by far more important than heating by neutral reactive particles such as atoms, especially for small powder particles. Here, it should be stressed that Equation (9) is just a rough, but a useful approximation.

Results of simple calculation, as presented above, reveal a huge difference between the heating of large objects (e.g., polymer foils) and small but macroscopic particles (e.g., polymer powder) in gaseous plasma. While large objects remain close to room temperature upon plasma treatment, small particles of dimension close to the Debye length or even smaller levitating in gaseous plasma heat significantly. The smaller is the particle diameter, the larger is the thermal load. This observation is a consequence of sheath formation. The positively charged ions are collected on the surface area much larger than the diameter of a powder particle, so the heating by ion bombardment and surface neutralization is extensive.

Here, it should be stressed that the above calculations were made using several assumptions and simplifications. First, the calculations of heating by ions are valid for the case the sheath thickness is much larger than the particle radius. A better approximation will be replacement of *r*_sheath_ in Equation (9) with the sum of *r*_sheath_ + *r*, but the weakly ionized plasma often used for treatment of polymer materials is of low density of charged particles so the sheath thickness should be larger than the radius of the polymer particle. Furthermore, the calculations are valid for collision-less sheaths. In real situation, there are always some collisions within the sheaths what makes calculations complicated. The collision path decreases with increasing pressure. At the pressure frequently used for experimental treatment of polymer powder (i.e., 100 Pa) the mean free path of gaseous molecules is roughly 0.1 mm, what is comparable to the sheath thickness in rather weakly ionized gaseous plasma. In fact, the Debye length of 0.1 mm develops in plasma of electron density 1.6 × 10^16^ m^−3^ at electron temperature of 3 eV [18]. Many plasma reactors useful for treatment of polymer materials with low-pressure gaseous plasma operate at such an electron density and temperature so the sheaths are not really collision-less. Equation (9) is void for the case polymer powder is treated in an afterglow of low-pressure plasma where the electron density is so low that the sheath thickness is approaching infinity.

The lower limit of plasma functionalization of polymer powder is defined by the fluence of reactive species onto the treated samples. Unfortunately, there is no report on recommended fluences for saturation of polyethylene surfaces with polar functional groups, but Vukusic et al. reported the optimal wettability of acryl-coated polypropylene foils after receiving the fluence of about 2 × 10^22^ O-atoms/m^2^ [19]. Similar numbers were also reported by Vesel et al. for the case of polystyrene [20].

## 3. Materials and Methods

The results of simple calculations presented in Section 2 were considered when designing experiments for plasma functionalization of PE powders. Instead of levitating powder in gaseous plasma, we performed experiments in a dish exposed to diffusing plasma of low ion density and high density of oxygen atoms in the ground state, stirring the powder upon plasma treatment and thus preventing overheating.

### 3.1. Materials

Commercially available PE powder was supplied by Dow corporate (Midland, Michigan). Product name: Dowlex™ 2629.10UE Polyethylene Resin. The type of polymer was hexane-based linear low-density polyethylene (LLDPE) with melt index 3.8 g/min (ASTM D1238—standard test method for melt flow rates of thermoplastics by extrusion plastometer), density 0.9370 g/cm^3^ (ASTM D792—Standard test methods for density and specific gravity (relative density) of plastics by displacement) and bulk density 0.346 g/cm^3^ (ASTM D1895—standard test methods for apparent density, bulk factor and pourability of plastic materials). Particle size was determined by Vibratory Sieve Shaker AS 200 basic with test sieves of 300, 250, 200, 150, 100, 63 and 40 μm (Retsch, Haan, Germany) via creating of cumulative curve, where d_50_ = 160 µm, d_10_ = 64 µm and d_90_ = 246 µm. This material is designed for rotational and injection molding with application in intermediate bulk containers, drums for chemicals, boats, freezer containers, etc. [21]. No pretreatment was performed before plasma treatments.

### 3.2. Plasma Reactor

**Figure 3:**
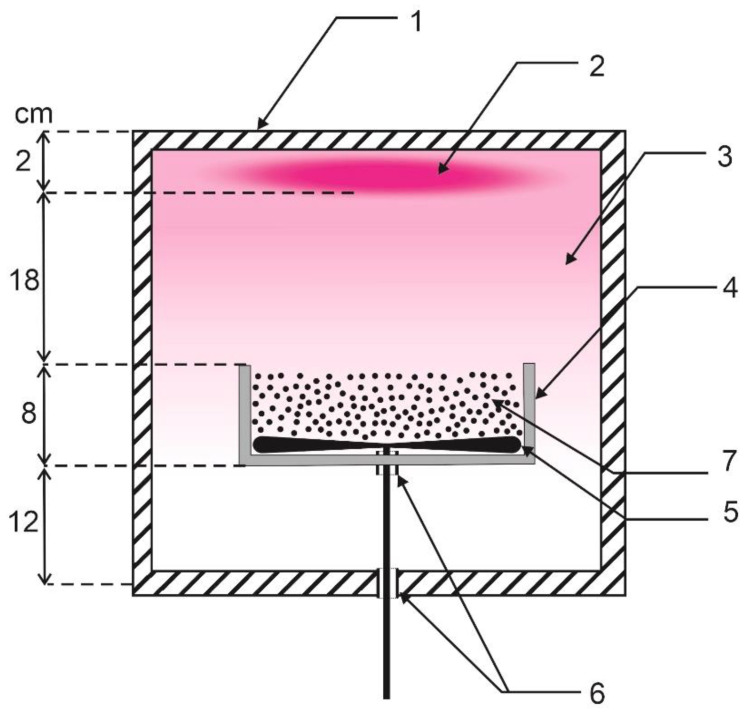
Schematic of the plasma reactor: (**1**) cubicle reactor with dimensions 40 × 40 × 40 cm^3^; (**2**) dense plasma; (**3**) diffusing plasma; (**4**) fixed stainless–steel dish; (**5**) rotatable stirring device; (**6**) rotatable troughs; and (**7**) polymer powder.

Samples were treated in a semi-industrial reactor for the treatment of polymer powders LA400 produced by Surface-Treat Ltd. (Turnov, Czech Republic). Details of the reactor are given elsewhere [14]. Briefly, the reactor is a rectangular vacuum vessel of a volume of 64 L made from aluminum. The reactor is pumped with a two-stage rotary pump of a nominal pumping speed of 65 m^3/^h (Duo 65, Pfeiffer Vacuum, Asslar, Germany). The oxygen of commercial purity is leaked into the plasma reactor upon continuous pumping. The leak rate was adjusted using variable flow controllers (MKS Instruments, type 1179BX22CM1BV, Munich, Germany). The flow of oxygen was fixed to 100 sccm for the experiments reported in this paper. There was a manually adjustable butterfly valve between the plasma reactor and the vacuum pump that allowed for adjustment of gas pressure inside the plasma reactor fairly independently from the pumping speed. The valve was adjusted to allow for keeping the pressure during plasma treatment of samples at 100 Pa. The pressure was measured with a calibrated Pirani gauge (TPR 280, Pfeiffer Vacuum, Asslar, Germany). Schematic of the discharge chamber is shown in Figure 3.

Plasma was sustained in the entire reactor using a pulsed microwave discharge powered with a magnetron source mounted on the top flange of the plasma reactor. The on-time was fixed to 70 μs and off-time was fixed to 130 μs. The magnetron allowed for sustaining rather dense and luminous plasma within a few centimeters from the upper flange. There was a diffusing plasma of low luminosity in the rest of the plasma reactor, as shown schematically in Figure 3. Powder samples were placed in a stainless-steel dish of cylindrical shape and the diameter and height of 20 and 8 cm, respectively. A stirring device of 40 rpm enabled mixing the polymer powder upon plasma treatment.

The density of oxygen atoms in the plasma reactor just above the dish filled with polymer powder was measured with a fiber-optics catalytic probe FOCP-2 provided by Plasmadis Ltd. (Ljubljana, Slovenia). The probe enabled real-time measurements with the temporal resolution of about 1 s and spatial resolution of 3 mm [22]. The accuracy of the probes is estimated at ±20%. The O-atom density during the powder treatment was almost perfectly constant at the value of 2 × 10^21^ m^−3^.

### 3.3. Wettability and Surface Characterization

The untreated and plasma-treated polymer powder was characterized to determine the wettability and the presence of polar functional groups. The wettability was probed by self-constructed machine based on tensiometer determined dynamic capillarity according to Washburne method [23]. The suction of powder material is determined by penetration of benzylalcohol at 25 °C. Values of suction [g^2^/s] are normalized to the value gained from untreated sample, which is defined as 100%.

The concentration of oxygen on the plasma-treated powder was measured by ESCA-3 Mk. II X-ray Photoelectron Spectroscopy (XPS) (Thermo VG Scientific, East Grinstead, UK). The measurement was done under the pressure of residual gases in the analyzer chamber of spectrometer 5 × 10^−10^ mbar. Monochromatic radiation Al K_α_ (hʋ = 1486.6 eV) was used for excitation of photoelectrons. The spectra in the range of binding energies 0–1000 eV were measured together with measurement of difference line C 1s and O 1s in high resolution mode. The measurement error of binding energies difference was ±0.2 eV. The surface stoichiometry was calculated from integral intensities of photoemission lines after subtraction of Shirley background [24], correction to transmission function of used hemispherical analyzer of electrons and on theoretical Scofieldian values partial photoelectrons cross-sections [25]. Software XPSPEAK 4.1. (Raymond W.M. Kwok, Chinese University of Hong Kong, Hong Kong, China) was used for fitting Gauss–Lorentzian functions. The usual error of the concentration values thus obtained is up to about ±10%.

## 4. Results and Discussion

Polymer powder of 250 g per batch was poured into the stainless-steel dish, which was equipped with a simple device for stirring. The plasma reactor was pumped down to the ultimate pressure of few Pa. The pump-down time was about 100 s. When the ultimate pressure was reached, the flow-controller was adjusted to 100 sccm and the butterfly valve in such a way to assure for the pressure of 100 Pa. The stirring device and the magnetron plasma source were turned on, and the polymer powder was treated at the constant MW power of about 1 kW. The reactor was vented after the treatment, and the polymer powder was characterized. The selected treatment times were 0.5, 1, 3, 5, 10 and 20 min. The temperature of the stainless-steel dish slowly increased with increasing treatment time but remained below 50 °C, even for the longest treatment time of 20 min. The rather poor heating of the dish with the PE powder is explained by the fact that the dish was placed in the center of the plasma reactor where only the diffusing plasma was presented, and the rather large thermal capacity of the dish was filled with the polymer powder. As mentioned above, the O-atom density just above the dish was about 2 × 10^21^ m^−3^. Because the neutral gas kinetic temperature above the dish is not much different from the room temperature, the flux of atoms onto the surface of the polymer powder in the dish according to Equation (1) was: *j*_N_′ = ¼ 2 × 10^21^ m^−3^ × 630 m/s ≈ 3 × 10^23^ m^−2^ s^−1^. Replacing *S*_powder_ in Equation (2) with the surface of the stainless-steel dish *S*_dish_ = 0.03 m^2^, one can estimate the heat flux (power dissipated on the surface of the polymer powder in the dish) because of heterogeneous surface recombination to P′ = 3 × 10^23^ m^−2^ s^−1^ × 0.03 m^2^ × 0.001 × 4 × 10^−19^ J ≈ 4 W. This power is small enough to assure for minimal heating of the dish filled with PE powder. Unfortunately, the density of charged particles was not measured; therefore, it was not possible to estimate the heating caused by charged particles. However, because the plasma density was low at the position of the dish, and the Debye length much smaller than the dish surface, the heating by ions cannot be significant.

**Figure 4:**
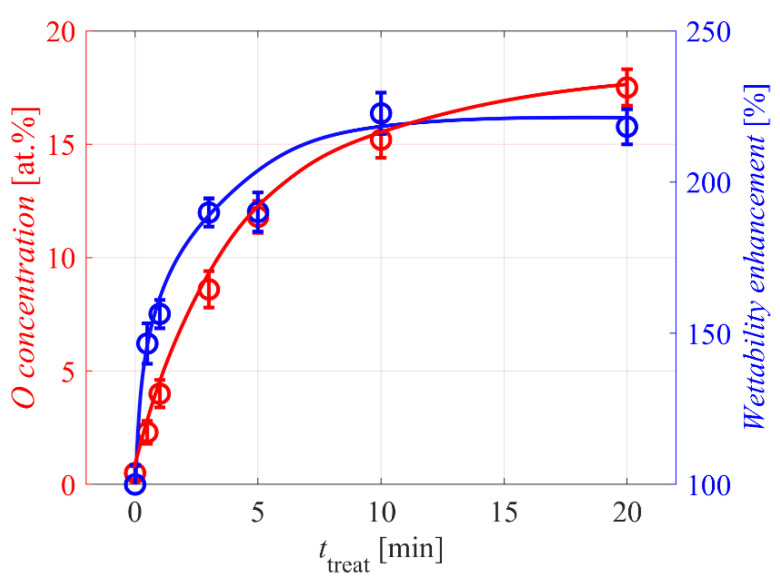
Oxygen concentration and increased wettability versus plasma treatment time.

The composition of the surface film, as probed by XPS, was deduced from survey spectra. As expected, only carbon and oxygen were found for all samples. The concentration of both elements, however, depended on the treatment time. Figure 4 represents the oxygen concentration in the surface film as probed by XPS versus the treatment time. The concentration of oxygen on the surface of untreated PE powder was at the edge of the detection limit of XPS—about 0.5 at.%. Even half a minute of exposure to gaseous plasma caused an increase in the oxygen concentration to about 2.3 at.%. Prolonged treatment caused further functionalization of the PE powder. The increase of the oxygen concentration, as revealed in Figure 4, was gradual but not linear. This observation is explained by extensive functionalization for the first few minutes, followed by a saturation effect. Namely, the number of binding sites on the non-functionalized polymer surface is limited. The concentration of oxygen reached after 20 min of plasma treatment is 17.5 at.%. This value is typical for well-functionalized PE foils [4,5,6]; therefore, one can conclude that the powder surface was saturated with oxygen functional groups after the 20-min treatment.

**Figure 5:**
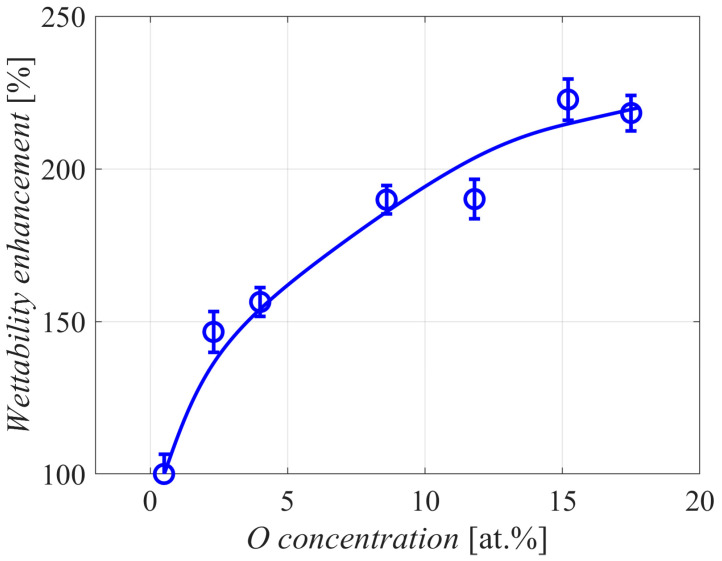
The increased wettability versus the oxygen concentration on the surface of PE powder.

The increase of wettability versus the treatment time is also plotted in Figure 4. Even the shortest treatment time of 0.5 min enabled a significant increase to about 150% of the original value, as determined by the Washburne method. The wettability curve in Figure 4 follows the curve for oxygen concentration but is much steeper for short treatment times. This observation indicates that the surface of the polymer powder does not have to be saturated with oxygen groups in order to benefit from improved wettability. Even a few percent of oxygen on the polymer surface causes a significant increase in the wettability, indicating non-linear behavior. The effect is best examined in Figure 5, which shows the wettability versus the oxygen concentration as probed by XPS. The curve is not linear but rather parabolic. This observation is different from the results reported in [9] for the case of PE powder treated in a fluidized bed reactor, where a linear relationship was observed. The discrepancy may be due to the thermal effects likely to occur in such experimental setups.

The functionalization efficiency can be estimated from the measured density of neutral oxygen atoms in the plasma reactor and the surface of the polymer powder, taking into account the fluence needed for saturation of the polymer surface with oxygen functional groups from the available literature, i.e., 2 × 10^22^ O-atoms/m^2^ [19,20]. The required treatment time for receiving this fluence depends on the surface of all the powder particles Σ*S*_powder_, the surface of the stainless–steel dish *S*_dish_ and the flux of O-atoms on the surface of the dish:

(10) $$\tau =\frac{\left(\frac{\Sigma S_{\mathrm{powder}}}{S_{\mathrm{dish}}}\right)\cdot D}{j_{N}}$$

The real surface of each powder particle depends on the roughness and is difficult to estimate. In the first approximation, the particles are taken as spheres of a perfectly smooth surface. The surface of spherical particles of radius *r* in polymer powder of mass *M* is

(11) $$\Sigma S_{\mathrm{powder}}=\frac{3M}{\rho \cdot r}$$

where *M* is the mass of PE powder in a batch (250 g in our case). For polymer particles of radius 80 µm, the Σ*S*_powder_ ≈ 10 m^2^ and the time needed for receiving the fluence of 2 × 10^22^ O-atoms/m^2^ is τ = 21 s.

Figure 4 reveals the optimal wettability after the treatment for about 10 min. This is about 30-times longer treatment time than calculated from Equation (10). The discrepancy is easily attributed to the rich morphology of the PE powder because the required treatment time was calculated by considering the perfect smoothness of spherical particles. Polymer powder is rarely perfectly spherical and smooth; therefore, by using Equation (11), we underestimated the effective surface and correspondingly the treatment time needed for optimal wettability.

Another feasible explanation for the discrepancy between the observed surface effects as in Figure 3 and the calculated optimal treatment time as in Equation (10) is inadequate mixing of the polymer powder. The stirring of the powder does not assure for optimal positioning of dust particles to be treated by plasma radicals. However, the discrepancy is roughly an order of magnitude. By considering the underestimations because of non-realistic effective dust surface, it is possible to conclude that the device used for plasma treatment of polymer dust in this study may not be optimal, but it is not too far from the configuration that enables rather uniform treatment of PE powder at minimal energy consumption and on a semi-industrial level.

The plasma-treated polymer powder is useful for a better adhesion of polymer on various substrates. For example, any product that has to be covered with a thin polymer film should be coated with plasma-treated powder to ensure for compatibilities of surface properties, and then heated to the appropriate temperature to melt the polymer. The plasma-treated powder will assure for good adhesion of the polymer film on the surface of the product. Even more important application is for synthesizing polymer composites. Nowadays, many plastics are actually composites of inorganic and/or organic particles embedded in the polymer matrix. The plasma treatment of powder will assure for better adhesion between the particles and the polymer and thus improved mechanical properties of the final product

## 5. Conclusions

The experiments and simple calculations provided in this paper indicate the usefulness of stirring polymer powder upon treatment with weakly ionized oxygen plasma of moderately high dissociation fraction. The rather large O-atom density in the reactor enabled improved wettability already after a minute of plasma treatment. Thermal effects were suppressed by interacting the diffusing plasma with the polymer powder rather than gaseous plasma sustained in the region of high electromagnetic field provided by the pulsed magnetron source. The saturation of both wettability and surface functionalization was observed after about 20 min of plasma treatment at the specific experimental conditions. By measuring the density of O atoms, it was possible to estimate the treatment efficiency. The results enable upscaling of the technique to larger systems taking into account the experimentally determined values of O-atom density, treatment time, amount of polymer powder and stirring conditions.
